# Exploratory analysis of serum HER2 extracellular domain for HER2 positive gastric cancer treated with SOX plus trastuzumab

**DOI:** 10.1007/s10147-024-02509-z

**Published:** 2024-04-08

**Authors:** Takeru Wakatsuki, Naoki Ishizuka, Shuichi Hironaka, Keiko Minashi, Shigenori Kadowaki, Masahiro Goto, Hirokazu Shoji, Hidekazu Hirano, Izuma Nakayama, Hiroki Osumi, Mariko Ogura, Keisho Chin, Kensei Yamaguchi, Daisuke Takahari

**Affiliations:** 1 Department of Gastrointestinal Medical Oncology, The Cancer Institute Hospital of Japanese Foundation for Cancer Research, 3-8-31 Ariake, Koto-Ku, Tokyo, 135-8550 Japan; 2 Department of Clinical Trial Planning, The Cancer Institute Hospital of Japanese Foundation for Cancer Research, Tokyo, Japan; 3 Clinical Trial Promotion Department, Chiba Cancer Center, Chiba, Japan; 4 Department of Medical Oncology, Faculty of Medicine, Kyorin University, Tokyo, Japan; 5 Department of Clinical Oncology, Aichi Cancer Center Hospital, Nagoya, Japan; 6 Cancer Chemotherapy Center, Osaka Medical and Pharmaceutical University Hospital, Osaka, Japan; 7 Department of Gastrointestinal Medical Oncology, National Cancer Center Hospital, Tokyo, Japan

**Keywords:** HER2, Serum HER2 ECD, Gastric Cancer, Trastuzumab, Biomarker

## Abstract

**Background:**

The aim of this study was to explore the clinical utility of serum HER2 extracellular domain (sHER2 ECD) using data from a clinical trial evaluating trastuzumab combined S-1 plus oxaliplatin (SOX**)** in HER2 positive gastric cancer.

**Methods:**

sHER2 ECD were prospectively measured at baseline and subsequent treatment courses. Based on each quantile point of baseline sHER2 ECD levels and its early changes, patients were divided into two groups and compared clinical outcomes.

**Results:**

43 patients were enrolled, and 17 patients (39.5%) were positive for baseline sHER2 ECD. Higher baseline sHER2 ECD levels tended to have lower hazard ratios (HRs). When divided into two groups by baseline sHER2 ECD of 19.1 ng/ml, median progression-free survival (PFS) and overall survival (OS) was longer in the higher group (mPFS: 16.8 vs 8.7 months, *p* = 0.359. mOS: 35.5 vs 20.6 months, *p* = 0.270), respectively. After initiation of treatment, sHER2 ECD significantly decreased up until the third cycle. Higher reduction rates of sHER2 ECD within 3 cycles also tended to have lower HRs. When divided into two groups by reduction rate of 42.5%, mPFS and mOS was longer in the higher reduced group (mPFS: 17.2 vs 8.7 months, *p* = 0.095. mOS: 65.0 vs 17.8 months, *p* = 0.047), respectively. Furthermore, higher reduction rates could surrogate higher objective response rates (ORR) (ORR: 90% vs 63.2% for 29.5%, *p* = 0.065. 100% vs 70% for 42.5%, *p* = 0.085), respectively.

**Conclusions:**

Baseline sHER2 ECD levels and its early decline may be useful biomarkers for SOX plus trastuzumab efficacy in HER2 positive gastric cancer.

**Supplementary Information:**

The online version contains supplementary material available at 10.1007/s10147-024-02509-z.

## Introduction

Human epidermal growth factor 2 (HER2) is one of the famous oncogenes in breast and gastric cancer [1]. HER2 overexpression or its gene amplification have been reported up to 20% in gastric cancer [2–4], suggesting that it is a critical driver oncogene and a promising treatment target. Trastuzumab, a humanized monoclonal anti-HER2 antibody, has been successfully developed in HER2 positive breast cancer [5, 6]. In gastric cancer, the ToGA trial showed significant clinical benefits of the addition of trastuzumab to standard chemotherapy [7]. Therefore, trastuzumab should be used as the first-line treatment for HER2 positive gastric cancer. More recently trastuzumab deruxtecan, an antibody − drug conjugate consisting of trastuzumab covalently linked to topoisomerase I inhibitor, has been approved in HER2 positive gastric cancer [8]. However, not all patients benefit from these anti-HER2 agents; therefore, useful biomarkers which predict treatment efficacy are needed.

HER2 is a 185 kDa glycoprotein consisting of three domains: a 105 kDa extracellular domain (ECD), a transmembrane domain, and an intracellular domain. HER2 ECD is shed into the circulation from tumor surface by proteolytic cleavage, and it can be detected in serum [9]. Preclinical studies suggested that serum HER2 ECD (sHER2 ECD) can bind to anti-HER2 antibody: neutralizing the biological effect of trastuzumab [10]. Cleavage of HER2 ECD also leaves a truncated form of HER2 (p95 HER2) which shows continuous kinase activity without dimerization, suggesting potential mechanism of primary or acquired resistance to trastuzumab [11, 12]. On the other hand, there have been many reports suggesting clinical utility of sHER2 ECD in breast cancer: elevated sHER2 ECD level at baseline or its early decline after trastuzumab treatment were associated with better response and survival in trastuzumab-based chemotherapy [13–17], although not all reports were consistent [18, 19]. In gastric cancer, there is limited data on the clinical utility of sHER2 ECD [20–22]. Moreover, no prospective study has been done in HER2 positive gastric cancer; therefore, clinical significance of sHER2 ECD on trastuzumab-based chemotherapy should be elucidated. Previously, we conducted clinical trial evaluating trastuzumab combined S-1 plus oxaliplatin (SOX) in HER2 positive gastric cancer [23]. Biomarker analysis was run alongside this clinical trial. The aim of this study was to explore the clinical utility of baseline sHER2 ECD levels and its early changes in trastuzumab plus SOX for HER2 positive gastric cancer. In addition, we investigated clinicopathological features related to baseline sHER2 ECD level.

## Patients and methods

### Patients

A phase II study of Trastuzumab with S-1 plus Oxaliplatin for HER2 positive advanced gastric cancer (Herceptin® with SOX for Gastric Cancer; HIGHSOX) study was a multicenter, prospective, single arm, phase II trial. The primary endpoint was the confirmed overall response rate according to the Response Evaluation Criteria in Solid Tumors (RECIST) v1.1 guideline. Patients with histologically confirmed, chemo-naïve gastric or esophagogastric junction adenocarcinoma with measurable lesions according RECIST v 1.1, and HER2-positive status determined by immunohistochemistry (IHC) and fluorescence in situ hybridization (FISH) (IHC 3 + or IHC2 + and FISH positive) were enrolled. More detail information is described in elsewhere [23]. Patients who participated in the HIGHSOX study and gave consent were enrolled in this biomarker study.

### Treatment

Patients received the treatment every three weeks until disease progression or unacceptable toxicity. Trastuzumab (course 1, 8 mg /kg; course 2-, 6 mg/kg) and oxaliplatin (130 mg/m^2^) intravenously infusion on day 1 and S-1 twice a day orally at a dose based on body surface area (< 1.25 m^2^, 40 mg; 1.25–1.5 m^2^, 50 mg; > 1.5 m^2^, 60 mg) on days 1–14 of a 21-day cycle. Tumors were measured every 6 weeks by the investigators at each participating center until progressive disease. Complete response and partial response were confirmed 4 weeks after the initial assessment. An Independent Data Monitoring Committee reviewed all efficacy and safety data.

### Samples and assay methods

Sample collection and measurement of sHER2 ECD were prospectively conducted each treatment course. Blood samples were collected in a collection tube with coagulation promoter and separating agent at before initial treatment and subsequently before each treatment course. After collection, the samples were inverted sufficiently and stood for 30 min. Then, samples were centrifuged at 3000*g* for 10 min under refrigeration. The obtained serum samples were sent to SRL, Inc. Tokyo, Japan. sHER2 ECD levels were prospectively measured by chemi-luminescence immunoassay using anti-HER2 monoclonal antibodies. Upper limit of normal of HER2 ECD level was 15.2 ng/ml in this assay [24].

### Ethics statement

The study was performed in accordance with the Declaration of Helsinki and Ethical Guidelines for Clinical Studies in Japan. The protocol was approved by the ethics committees of The Cancer Institute Hospital and of each participating center before initiating enrollment (2015–1027). All patients provided written informed consent.

### Statistical analysis

Since sHER2 ECD levels were widely different among patients, normal values of sHRE2 ECD converted to base 10 logarithmic values, and subsequent analysis was performed. Associations between baseline sHER2 ECD level and clinicopathological factors listed in patients’ characteristics in Table 1 were assessed; Mann − Whitney *U* test was performed in categorical valuables and Spearman’s rank correlation coefficient was calculated in continuous variables. Covariates whose *p* value was less than 0.05 were included in multiple regression analysis. Comparisons in sHER2 ECD levels between baseline and subsequent treatment courses were done by Dunnett − Hsu test.

**Table 1:** Pagtients' characteristics

	*N* = 43 (%)
Age	
Median (Range)	64 (21–75)
Gender	
Male	35 (81.4)
Female	8 (18.6)
ECOG PS	
0	35 (81.4)
1	8 (18.6)
Primary Site	
Stomach	35 (81.4)
GEJ	8 (18.6)
Histology	
Differentiated	25 (58.1)
Undifferenciated	18 (41.9)
Previous gastrectomy	
Yes	8 (18.6)
No	35 (81.4)
No. metastatic organs	
Median (Range)	1 (1–5)
Metastatic Site	
Liver	21 (48.8)
Lymph node	18 (41.9)
Peritoneum	9 (20.9)
Sum of diameter in target lesions (mm)	
Median (Range)	43 (12–183)
HER2 status	
IHC 3 +	35 (81.4)
IHC 2 + /FISH positive	8 (18.6)
Serum HER2 ECD (ng/ml)	
Median (Range)	12.8 (9.2–3440.0)
CEA (ng/ml)	
Median (Range)	11.2 (0.8–10,064.5)
CA19-9 (U/ml)	
Median (Range)	108.0 (2–507,479.0)

*CEA* Carcinoembryonic antigen, *CA19-9* Carbohydrate antigen 19-9, *ECD* Extracellular Domain, *ECOG PS* Eastern Cooperative Oncology Group Performance Status, *GEJ* Gastroesophageal junction, *IHC* immunohistochemistry, *FISH* fluorescence in-situ hybridization

Progression-free survival (PFS) was defined as the time from the first day of treatment to either the first objective evidence of disease progression or death from any cause. The overall survival (OS) was defined as the time from the first day of treatment until the time of death. For this analysis, we extended data follow-up until the end of 2023. The objective response rate (ORR) was evaluated according to RECIST v1.1. To evaluate prognostic factors in this cohort, PFS and OS were estimated using the Kaplan − Meier methods and compared by the log-rank test. Multivariate analysis using Cox proportional hazard model was performed including covariates whose *p* value was less than 0.05 in univariate analysis. In multivariate analysis, we defined an independent prognostic factor whose *p* value was less than 0.05. In comparisons of ORR, Chi-square test or Fisher’s exact test were performed.

This was an exploratory study to evaluate the clinical significance of the baseline sHER2 ECD level and its early change on trastuzumab-based chemotherapy. To find the optimal cutoff value, cutoff values were set based on the quantile points of base line sHER2 ECD level and its early change. PFS and OS were dichotomized at these cutoff values and compared. The optimal cutoff value was chosen based on the lowest hazard ratio point estimate. We defined early change as the greatest reduction rate in sHER2 ECD between baseline and until the third courses. Survival and ORR were compared by each cutoff value. All statistical tests provided two-sided *p* values, with *p* < 0.05 considered significant. All statistical analysis was done using the SPSS ver.24 (SPSS Chicago, IL).

## Results

### Patients’ characteristics

Seventy-five patients participated in the HIGHSOX study. Among them, 43 patients were enrolled in this biomarker study and were measured baseline sHER2 ECD level. Patients` characteristic in this study are shown in Table 1. The median age was 64 years old, and around 80% of patients were male, ECOG PS0, stomach primary site, no previous gastrectomy, and HER2 IHC status 3 + . Almost 50% of patients had liver metastasis, and distant lymph node metastasis and peritoneal metastasis were found in more than 40% and 20% of patients, respectively. The median sum of the longest diameters of target lesions was 43 mm in this cohort. Patients` characteristic in this study was comparable to those of the HIGHSOX study [23].

### Baseline HER2 ECD level and clinicopathological factors

**Fig. 1:**
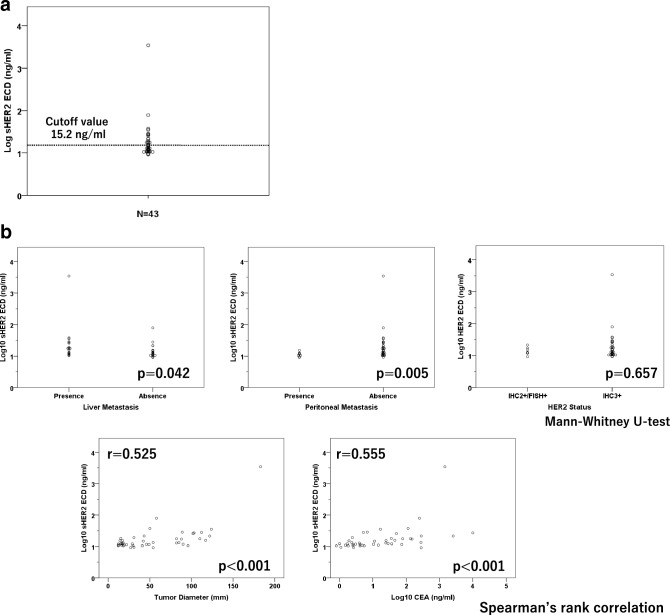
**a** Distributions of baseline serum HER2 ECD levels are shown. Normal values of serum HRE2 ECD were converted to base 10 logarithmic values. **b** Associations between patients’ characteristics and baseline serum HER2 ECD levels are shown. Several characteristics were significantly associated with baseline serum HER2 ESD levels, but HER2 status was not

Distribution of baseline sHER2 ECD levels is shown in Fig. 1a. The median baseline sHER2 ECD level was 12.8 ng/ml (Range 9.2–3440.0 ng/ml, Interquartile range; 11.1–19.1 ng/ml) with positivity rate of 39.5% in this study. Among clinicopathological characteristics, liver metastasis was significantly associated with higher baseline sHER2 ECD levels, while peritoneal metastasis was significantly associated with those of lower levels (Fig. 1b). No significant difference was shown in HER2 status (IHC3 vs IHC2 + /FISH +). In addition, a sum of the diameter in target lesions and baseline CEA levels showed significantly positive correlation with baseline sHER2 ECD levels. Multiple regression analysis including these factors showed that the sum of the diameter in target lesions was an only clinicopathological factor significantly related to baseline sHER2 ECD levels (Table 2).

**Table 2:** Clinicopathological Features and Baseline Serum HER2 ECD Level

Factors	Coefficient	*p* value
Liver metastasis	0.103	0.503
Peritoneal metastasis	0.204	0.88
Sum of diameter in target lesions	0.646	< 0.001
HER2 status	0.051	0.731
CEA levels	0.024	0.865

*ECD* Extracellular Domain, *CEA* Carcinoembronic Antigen

### Baseline HER2 ECD level and treatment efficacy

The median follow-up period of all patients was 26.5 months, and the median follow-up period of censored patients was 79.3 months. The median PFS and OS of patients in this biomarker study was 11.3 months and 26.5 months, respectively, with ORR of 72.1%. These results were like those in the HIGHSOX study (Online Resource 1–3). Peritoneal metastasis and HER2 IHC2 + /FISH positive were chosen as a negative prognostic factor by univariate analysis in PFS. However, these factors did not remain after multivariate analysis. As for OS, stomach tumor, number of metastatic organs ≥ 2, distant lymph node metastasis and peritoneal metastasis were chosen as a negative prognostic factor by univariate analysis in OS. In multivariate analysis, distant lymph node metastasis and peritoneal metastasis remained as an independent negative prognostic factor for OS (Table 3).

**Table 3:** Patients' Characteristics and Prognostic Factors in This Cohort

	mPFS	Univariate		Multivariate		mOS	Univariate		Multivariate			
		HR	*p*-value	HR	*p*-value		HR	*p*-value	HR		*p*-value	
*Age*												
< 64 (*n* = 21)	11.0(5.2–16.8)	Ref				26.8(17.8–35.9)	Ref					
≧64 (*n* = 22)	14.5(6.3–22.6)	1.08(0.56–2.06)	0.821			17.3(1.2–33.4)	1.27(0.65–2.47)	0.483				
*Gender*												
Male (*n* = 35)	11.0(4.1–17.8)	Ref				26.8(13.6–40.1)	Ref					
Female (*n* = 8)	11.8(0–26.1)	0.88(0.39–2.01)	0.765			16.9(7.0–26.8)	0.91(0.38–2.21)	0.841				
*ECOG PS*												
0(*n* = 35)	11.0(3.9–18.0)	Ref				26.8(13.6–40.1)	Ref					
1(*n* = 8)	11.3(0.0–24.2)	1.37(0.62–3.01)	0.441			14.5(0.0–34.5)	1.84(0.82–4.13)	0.140				
*Prior gastrectomy*												
Yes(*n* = 8)	6.7(0.0–14.8)	Ref				23.7(7.1–40.2)	Ref					
No(*n* = 35)	11.3(4.5–18.2)	1.32(0.55–3.19)	0.539			26.5(12.7–40.4)	1.18(0.49–2.83)	0.718				
*Tumor location*												
GEJ(*n* = 8)	11.0(2.9–19.1)	Ref				NR	Ref			Ref		
Stomach (*n* = 35)	11.3(0.0–47.3)	2.25(0.87–5.83)	0.096			17.3(10.6–24.0)	3.49(1.22–9.97)	0.020		2.89(0.97–8.61)		0.057
*Differentiation*												
Differentiated (*n* = 25)	14.7(8.0–21.4)	Ref				29.2(23.9–34.6)	Ref					
Undifferentiated(*n* = 18)	7.4(6.1–8.7)	1.37(0.71–2.63)	0.347			17.3(11.1–23.4)	1.35(0.69–2.64)	0.386				
*No. metastatic organs*												
0–1(*n* = 29)	14.5(8.0–21.0)	Ref				26.8(11.5–42.2)	Ref			Ref		
≧2(*n* = 14)	7.5(2.5–12.5)	1.90(0.95–3.80)	0.069			17.0(12.5–21.5)	2.26(1.11–4.60)	0.025		1.28(0.58–2.81)		0.542
*Tumor diameter*												
< 43 mm(*n* = 21)	11.0(6.5–15.5)	Ref				26.8(14.5–39.2)	Ref					
≧43 mm(*n* = 22)	11.3(3.0–19.6)	1.78(0.90–3.47)	0.098			17.8(3.7–31.8)	1.49(0.76–2.91)	0.245				
*Liver metastasis*												
Yes(*n* = 21)	11.3(2.2-.20.4)	Ref				29.2(11.3–47.2)	Ref					
No(*n* = 22)	11.0(2.9–19.0)	0.95(0.50–1.82)	0.881			20.6(6.8–34.4)	1.21(0.62–2.35)	0.581				
*Lymph node metastasis*												
Yes(n = 18)	11.0(7.0–14.9)	Ref				20.6(6.7–34.4)	Ref			Ref		
No(*n* = 25)	11.8(1.8–21.7)	0.75(0.39–1.43)	0.380			32.4(0.0–84.4)	0.48(0.24–0.97)	0.041		0.39(0.17–0.89)		0.025
*Peritoneal metastasis*												
Yes(*n* = 9)	6.7(3.5–9.8)	Ref		Ref		11.1(7.2–14.9)	Ref			Ref		
No(*n* = 34)	14.7(8.1–21.2)	0.43(0.20–0.93)	0.033	0.61(0.19–1.95)	0.402	29.2(22.2–36.3)	0.34(0.15–0.75)	0.007		0.31(0.13–0.76)		0.010
*HER2 status*												
IHC3 + (*n* = 35)	14.7(4.5–17.6)	Ref		Ref		29.2(16.6–41.9)	Ref					
IHC2 + /FISH + (*n* = 8)	5.9(3.8–8.0)	2.41(1.08–5.38)	0.031	1.65(0.49–5.52)	0.418	16.9(7.6–26.2)	2.13(0.94–4.81)	0.069				
*Baseline sHER2 ECD*												
< 15.2 (*n* = 26)	8.7(3.6–13.9)	Ref				23.7(8.7–38.7)	Ref					
≧15.2 (*n* = 17)	15.9(12.9–19.0)	0.76(0.39–1.48)	0.411			31.4(7.6–55.3)	0.72(0.36–1.43)	0.341				

*ECOG PS* Eastern Cooperative Oncology Group Performance Status, *IHC* Immunohistochemistry, *FISH* Fluorescence in-situ hybridization, *Baseline sHER2 ECD* Baseline serum HER2 extracellular domain

**Fig. 2:**
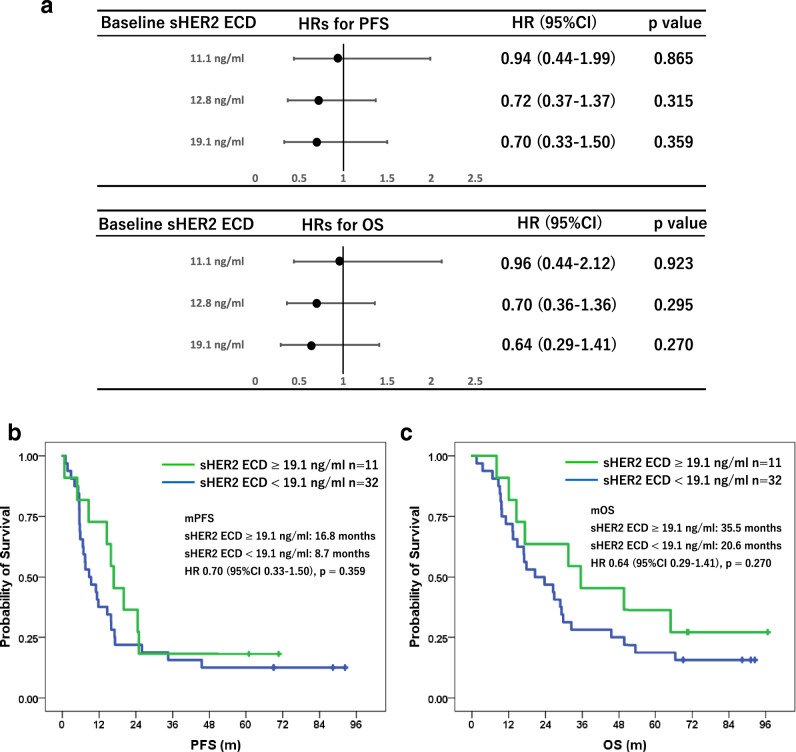
**a** Forrest plots show hazard ratios (HRs) by each quantile point of baseline serum HER2 ECD level. There was a trend toward lower HRs for higher baseline serum HER2 ECD levels both in PFS and OS, respectively. Kaplan–Meier curves of PFS **b** and OS **c** divided by baseline serum HER2 ECD with cutoff value of 19.1 ng/ml are shown, respectively

**Fig. 3:**
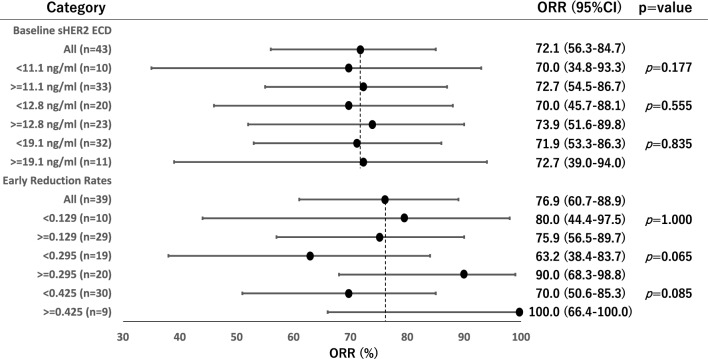
Forrest plots show objective response rates (ORR) by each quantile point of baseline serum HER2 ECD level and its early change. ORRs were comparable both in higher and lower baseline serum HER2 ECD groups even though divided by three selected cutoff values, while higher ORRs were shown in higher reduced group

The quartile points of baseline sHER2 ECD levels were 11.1 ng/ml at the 25th percentile, 12.8 ng/ml at the 50th percentile, and 19.1 ng/ml at the 75th percentile, respectively. PFS and OS were compared by each quantile point of baseline sHER2 ECD levels, and there was a trend toward lower HRs for higher baseline sHER2 ECD levels both in PFS and OS (Fig. 2a). Because the hazard ratio point estimate was smallest at the 75th percentile of 19.1 ng/ml for both PFS and OS, the optimal cutoff for baseline sHER2 ECD levels was set at 19.1 ng/ml. When divided into two groups by baseline sHER2 ECD level of 19.1 ng/ml, mPFS and mOS was longer in the higher baseline sHER2 ECD group compared with the lower group (mPFS: 16.8 months vs 8.7 months, HR 0.70 95%CI 0.33–1.50, *p* = 0.359. mOS: 35.5 months vs 20.6 months, HR 0.64 95%CI 0.29–1.41, *p* = 0.270), respectively (Fig. 2b, c). As for the ORR, ORRs were comparable both in higher and lower groups even though divided by three selected cutoff values (Fig. 3).

### Early changes in HER2 ECD during treatment and treatment efficacy

**Fig. 4:**
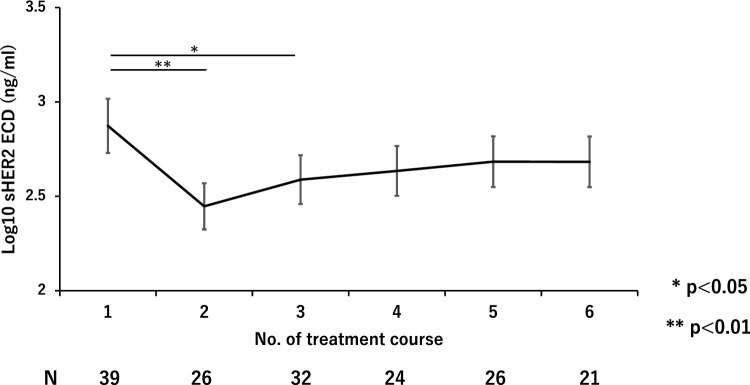
Changes in serum HER2 ECD during treatment are shown. After initiation of treatment, serum HER2 ECD had significantly decreased up until the 3rd cycle

**Fig. 5:**
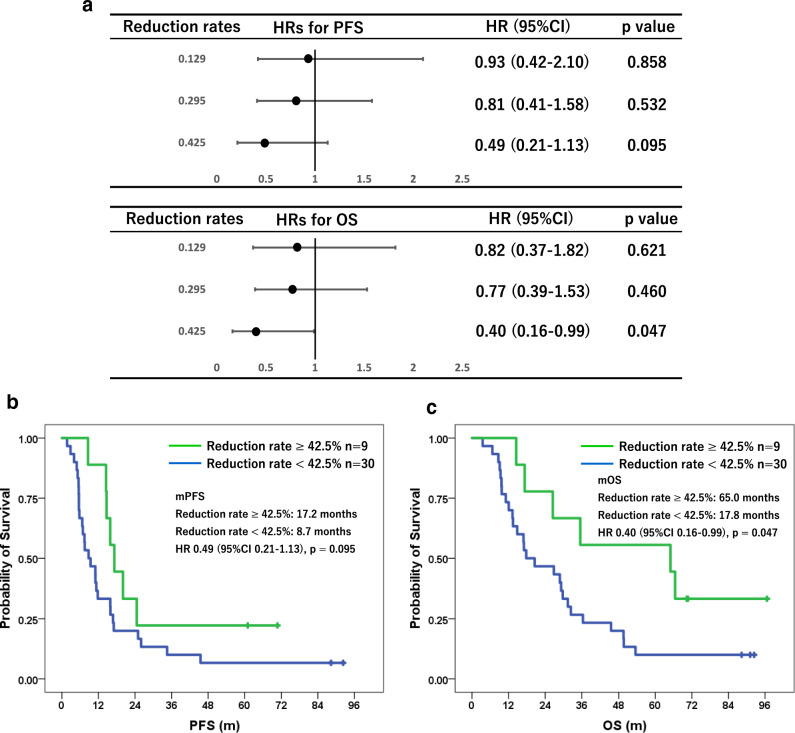
**a** Forrest plots show hazard ratios (HRs) by each quantile point of early changes in serum HER2 ECD level. There was a trend toward lower HRs for higher reduction rates of serum HER2 ECD both in PFS and OS, respectively. Kaplan–Meier curves of PFS **b** and OS **c** divided by reduction of 42.5% are shown, respectively. Patients who achieved a reduction rate of ≥ 42.5% within the first three treatment courses were significantly associated with longer OS

Among 43 patients, 39 patients were measured sHER2 ECD at least once within 3 cycles other than before their initial treatment. After initiation of treatment, sHER2 ECD had significantly decreased up until the third cycle (Fig. 4). Given that sHER2 ECD correlated with the sum of tumor diameter in target lesions and the significant decline in sHER2 ECD observed in the early after treatment, we next investigated the potential surrogate role of early sHER2 ECD reduction in treatment response. The quartile points of early changes in sHER2 ECD were 12.9% at the 25th percentile, 29.5% at the 50th percentile, and 42.5% at the 75th percentile, respectively. PFS and OS were compared by each quantile point of early changes in sHER2 ECD, and there was also a trend toward lower HRs for higher reduction rates of sHER2 ECD both in PFS and OS (Fig. 5a). Since the hazard ratio point estimate was smallest at the 75th percentile of 42.5% for both PFS and OS, the optimal cutoff for early changes in sHER2 ECD was set at 42.5%. When divided into two groups by reduction rate of 42.5%, mPFS and mOS were longer in the higher reduced group compared with the lower group (mPFS: 17.2 months vs 8.7 months, HR 0.49 95% CI 0.21–1.13, *p* = 0.095. mOS: 65.0 months vs 17.8 months, HR 0.40 95% CI 0.16–0.99, *p* = 0.047), respectively (Fig. 5b, c). As for the ORR, similar trends were shown when divided into two groups by reduction rates of 29.5% and 42.5%, ORRs were higher in the higher reduced group compared with the lower group (90% vs 63.2% for 29.5%, *p* = 0.065. 100% vs 70% for 42.5%, *p* = 0.085), respectively (Fig. 3).

Among 39 patients, 9 patients achieved the reduction rate ≥ 42.5% up until the third treatment courses. Among those 9 patients who achieved the reduction rate ≥ 42.5%, baseline sHER2 ECD levels were positive in 7 patients (77.8%) (Table 4). Whereas, among 30 patients who did not achieve the reduction rate ≥ 42.5%, baseline sHER2 ECD levels were negative in 22 patients (73.3%) (*p* = 0.015). Moreover, in nine patients who achieved this reduction rate up until the third treatment courses, 7 patients measured sHER2 ECD at the second cycle. Importantly, 6 out of 7 patients (85.7%) achieved the reduction rate ≥ 42.5% at the second cycle (3 weeks).

**Table 4:** Baseline sHER2 ECD positivity and achievement of reduction rate ≥ 42.5%

Baseline sHER2 ECD	Reduction rate ≥ 42.5% (*n* = 9)	Reduction rate < 42.5% (*n* = 30)	*p* value
Positive (*n* = 15)	7 (77.8%)	8 (26.7%)	0.015
Negative (*n* = 24)	2 (22.2%)	22 (73.3%)	

*sHER2 ECD* serum HER2 Extracellular Domain

## Discussion

In the context of the limited data on sHER2 ECD in HER2 positive gastric cancer, we obtained several findings from this study. First, the baseline sHER2 ECD levels were affected most by the sum of the diameter in target lesions. Second, there was a trend for higher baseline sHER2 ECD with better survival. Third, there were also tend for higher reduction rates both with better survival and response, respectively. Specifically, patients who achieved a reduction rate of ≥ 42.5% within the first three treatment courses were significantly associated with longer OS. Furthermore, most of these patients were baseline sHER2 ECD positive. This is the first prospective study describing the potential clinical utility of sHER2 ECD in trastuzumab efficacy in patients with HER2 positive gastric cancer.

According to previous reports, positivity rates of sHER2 ECD were 36–59% in metastatic HER2 positive gastric cancer [20, 21, 25–28]. Positivity rate of 39.5% in this study was in agreement with previous data, suggesting validity of this study. These previous reports have raised several clinicopathological factors affecting baseline sHER2 ECD levels: liver metastasis, clinical stage, primary tumor size, intestinal histology, and HER2 IHC status, reflecting distinct characteristics of HER2 positive gastric cancer [4]. Since these factors are thought to confound each other and based on the univariate analysis only, multivariate analysis is needed to identify true factors. Multiple regression analysis revealed that the sum of the diameter in target lesions was the only clinicopathological factor significantly affecting the baseline sHER2 ECD levels, suggesting that the baseline sHER2 ECD levels reflect tumor burden. Unlike previous reports, our data did not show a significant difference in terms of HER2 status [20, 24]. However, as reported previously, this study also indicates a higher distribution of sHER2 ECD in patients with IHC3 + (Fig. 1b), likely attributed to the small sample size in this study.

There have been several reports suggesting a correlation between baseline sHER2 ECD level and trastuzumab efficacy in HER2 positive gastric cancer [20–22]. Like the previous data, there was a trend toward lower HR for higher baseline sHER2 ECD levels in this study. Given that high tumor burden is generally considered as a negative prognostic factor, this result may seem contradictory at first glance. This discrepancy may be explained by the following reasons. Peritoneal metastasis was significantly associated with lower sHER2 ECD levels (Fig. 1b). Moreover, peritoneal metastasis was a negative prognostic factor in this study (Table 3). Therefore, patients with peritoneal metastasis would likely be skewed towards the lower baseline sHER2 ECD group. The baseline sHER2 ECD levels may represent a prognostic impact in HER2 positive gastric cancer rather than a predictive impact on trastuzumab efficacy.

sHER2 ECD significantly decreased during early treatment courses in this study. Likewise, another previous report supports our observation [22]. In addition, there was a trend toward lower HRs for higher reduction rates of sHER2 ECD. Specifically, patients who achieved a reduction rate of ≥ 42.5% within the first three treatment courses were significantly associated with longer OS. Moreover, a higher reduction rates of sHER2 ECD were correlated with higher ORRs in this study. Previous data also support clinical relevance between reduction rates in sHER2 ECD and trastuzumab efficacy [20, 21]. These consistencies suggest that early decline of sHER2 ECD can become a reliable surrogate marker for trastuzumab efficacy. As for surrogate marker, early tumor shrinkage (ETS), which was defined that ≥ 20% reduction in a sum of the diameter in target lesions after 8 weeks from initial treatment, is well known in gastrointestinal cancer [29–31]. Because early decline of sHER2 ECD (≥ 42.5%) was already observed at the 2 cycles (3 weeks) in most of the patients (85.7%) in this study, monitoring sHER2 ECD could be an even earlier predictor than ETS. In addition, early decline of sHER2 ECD (≥ 42.5%) was significantly found in patients whose baseline sHER2 ECD levels were positive (77.8% vs 22.2%, *p* = 0.015) (Table 4); monitoring sHER2 ECD would be useful particularly in patients whose baseline sHER2 ECD levels are positive. Further studies focusing on monitoring sHER2 ECD and ETS are warranted.

There are several limitations in this study. First of all, although this study is prospective multicenter study, sample size is too small to conclude regarding the clinical significance and optimal cutoff value for sHER2 ECD. Secondly, not all patients were measured sHER2 ECD every treatment cycle due to reimbursement issue because some patients were treated twice within the same month. Third, we do not have any nontrastuzumab data; we cannot distinguish whether these results are specific to trastuzumab-based chemotherapy. Finally, there is lack of molecular data such as intratumor HER2 heterogeneity [32, 33], HER2 DNA copy numbers [34], and cell free DNA [35–37], which have been proposed as a biomarker predicting trastuzumab efficacy. These limitations may keep a distance from firm conclusions and require further clinical validation using another larger cohort and molecular correlative analysis. Further studies using tissue samples are ongoing to see associations between sHER2 ECD and these factors.

## Conclusions

Baseline sHER2 ECD levels and its early decline may be useful biomarkers for SOX plus trastuzumab efficacy in HER2 positive gastric cancer. Monitoring sHER2 ECD should be considered for early prediction of better survival and treatment response, particular in patients with the positive baseline sHER2 ECD.

### Supplementary Information

Below is the link to the electronic supplementary material.

Supplementary file1 (PPTX 66 KB)

Supplementary file2 (PPTX 67 KB)

Supplementary file3 (XLSX 11 KB)
