# Supplementary figures and images for: Exploratory analysis of serum HER2 extracellular domain for HER2 positive gastric cancer treated with SOX plus trastuzumab
Source: Int J Clin Oncol. 2024 Apr 8;29(6):801–12. doi: 10.1007/s10147-024-02509-z (PMC11130043; doi:10.1007/s10147-024-02509-z)

## Slide 1
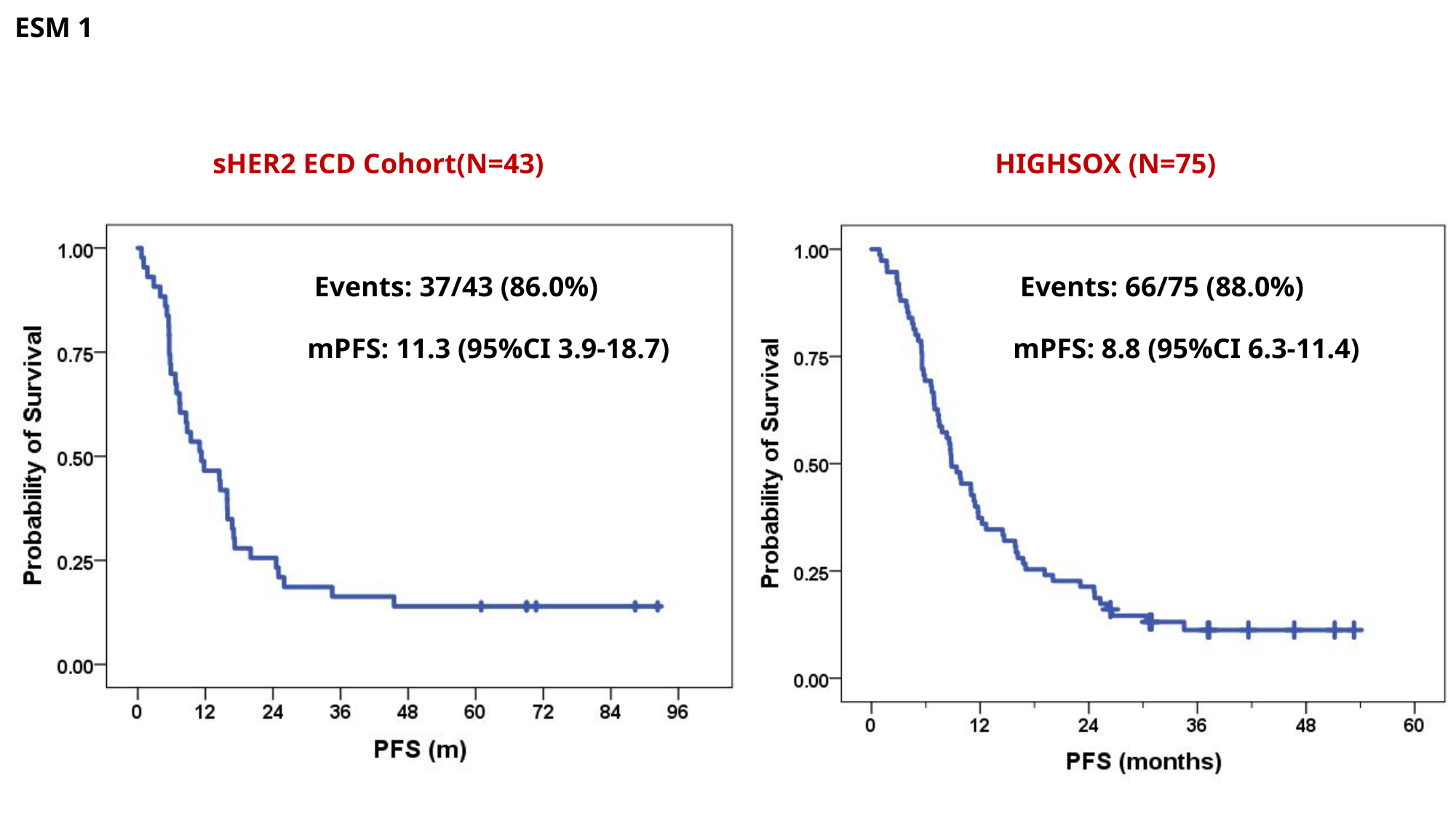

ESM 1
sHER2 ECD Cohort(N=43)
HIGHSOX (N=75)
Events: 37/43 (86.0%)
Events: 66/75 (88.0%)
mPFS: 11.3 (95%CI 3.9-18.7)
mPFS: 8.8 (95%CI 6.3-11.4)

Supplement: Supplementary file 1 — Supplementary file1 (PPTX 66 KB) [file 10147_2024_2509_MOESM1_ESM.pptx]

## Slide 1
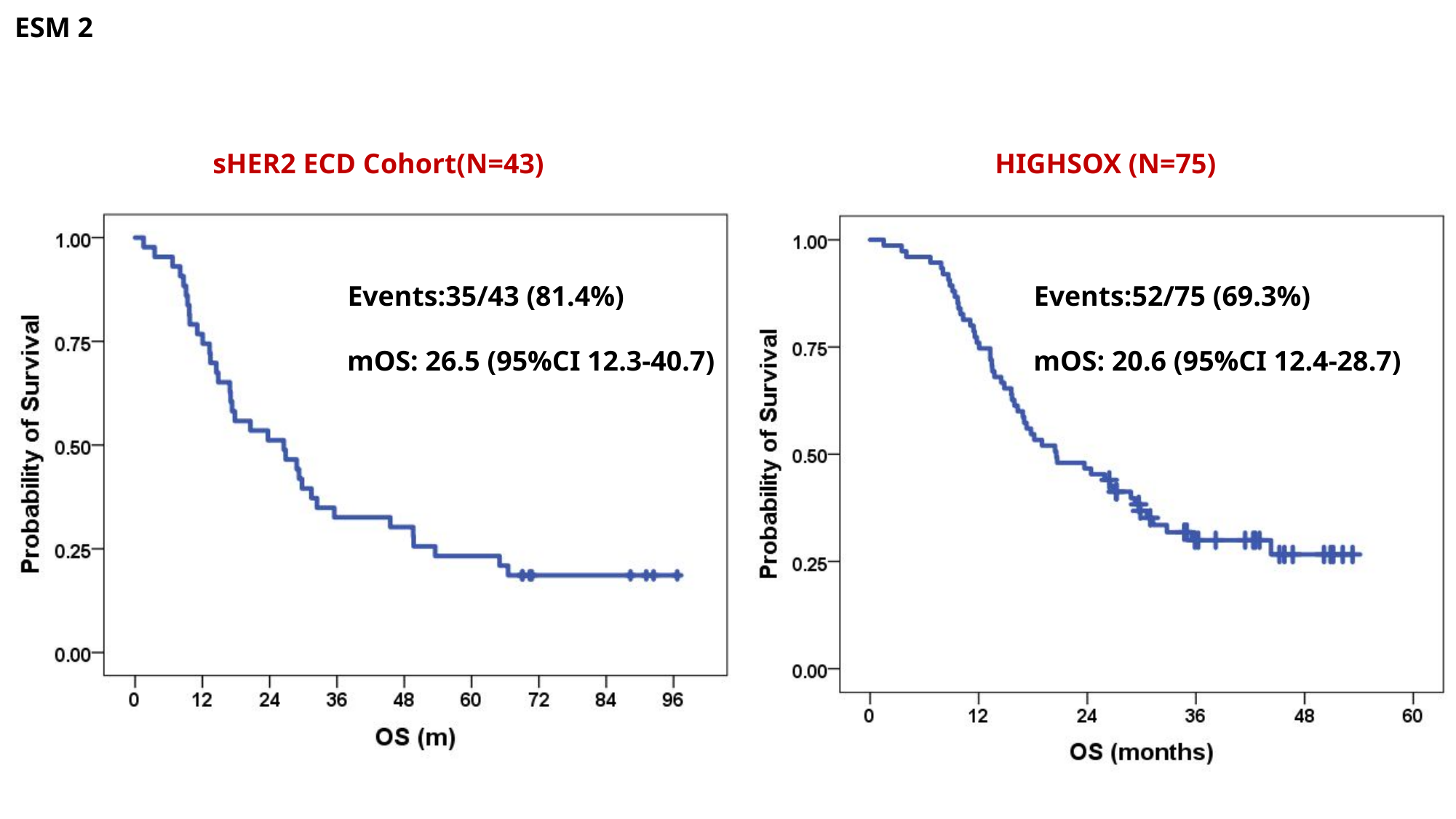

ESM 2
sHER2 ECD Cohort(N=43)
HIGHSOX (N=75)
Events:35/43 (81.4%)
Events:52/75 (69.3%)
mOS: 26.5 (95%CI 12.3-40.7)
mOS: 20.6 (95%CI 12.4-28.7)

Supplement: Supplementary file 2 — Supplementary file2 (PPTX 67 KB) [file 10147_2024_2509_MOESM2_ESM.pptx]
